# Expanding the molecular versatility of an optogenetic switch in yeast

**DOI:** 10.3389/fbioe.2022.1029217

**Published:** 2022-11-15

**Authors:** David Figueroa, Camila Baeza, Diego Ruiz, Claudia Inzunza, Andrés Romero, Rodrigo Toro, Francisco Salinas

**Affiliations:** ^1^ Laboratorio de Genómica Funcional, Facultad de Ciencias, Instituto de Bioquímica y Microbiología, Universidad Austral de Chile, Valdivia, Chile; ^2^ ANID–Millennium Science Initiative–Millennium Institute for Integrative Biology (iBIO), Santiago, Chile

**Keywords:** yeast, optogenetics, transcription, blue light, wine yeast

## Abstract

In the budding yeast *Saccharomyces cerevisiae*, the FUN-LOV (FUNgal Light Oxygen and Voltage) optogenetic switch enables high levels of light-activated gene expression in a reversible and tunable fashion. The FUN-LOV components, under identical promoter and terminator sequences, are encoded in two different plasmids, which limits its future applications in wild and industrial yeast strains. In this work, we aim to expand the molecular versatility of the FUN-LOV switch to increase its biotechnological applications. Initially, we generated new variants of this system by replacing the promoter and terminator sequences and by cloning the system in a single plasmid (FUN-LOV^SP^). In a second step, we included the nourseothricin (Nat) or hygromycin (Hph) antibiotic resistances genes in the new FUN-LOV^SP^ plasmid, generating two new variants (FUN-LOV^SP-Nat^ and FUN-LOV^SP-Hph^), to allow selection after genome integration. Then, we compared the levels of light-activated expression for each FUN-LOV variants using the luciferase reporter gene in the BY4741 yeast strain. The results indicate that FUN-LOV^SP-Nat^ and FUN-LOV^SP-Hph^, either episomally or genome integrated, reached higher levels of luciferase expression upon blue-light stimulation compared the original FUN-LOV system. Finally, we demonstrated the functionality of FUN-LOV^SP-Hph^ in the 59A-EC1118 wine yeast strain, showing similar levels of reporter gene induction under blue-light respect to the laboratory strain, and with lower luciferase expression background in darkness condition. Altogether, the new FUN-LOV variants described here are functional in different yeast strains, expanding the biotechnological applications of this optogenetic tool.

## 1 Introduction

Optogenetics technology began in neurobiology, where light-activated membrane transporters (bacterial channelrhodopsin-2) were used to control neurons upon light stimulation (Boyden et al., 2005; Deisseroth, 2011). After this pioneering experiment, the use of light as an activator of biological processes has grown considerably, largely due to its numerous advantages, including precise spatiotemporal resolution, moderated toxic effects, and the ability to replace the use of toxic and expensive chemical inducers (Shimizu-Sato et al., 2002; Wang et al., 2012). Optogenetics is based on photosensitive proteins known as photoreceptors, which are capable of light sensing at different wavelengths (Losi et al., 2018; Tang et al., 2021). Currently, optogenetic tools include a large set of different photoreceptors, which have been implemented in a variety of biological platforms (Kolar et al., 2018), including microorganisms such as the budding yeast *Saccharomyces cerevisiae* (Salinas et al., 2017; Figueroa et al., 2021).

Yeast is a prominent chassis for optogenetics due to the absence of photoreceptors encoded in its genome (Goffeau et al., 1996), enabling the use of light to orthogonally control biological processes. Furthermore, yeast is a well-known model organism for cellular and molecular biology studies, and a cell factory for the production of valuable proteins and metabolites (Nielsen, 2019). In yeast, optogenetics has proved its utility in the control of multiple cellular processes and biotechnologically relevant phenotypes, such as: gene expression, subcellular protein localization, protein-protein interaction, cell cycle, cell spatial organization, metabolic rewiring, and heterologous protein production (Shimizu-Sato et al., 2002; Tyszkiewicz and Muir, 2008; Witte et al., 2017; Xu et al., 2018; Figueroa et al., 2021; Moreno Morales et al., 2021; Hoffman et al., 2022).

Among the multiplicity of optogenetic tools developed in yeast, optogenetic switches enable light-controlled gene expression of any target gene (Salinas et al., 2017; Figueroa et al., 2021). Optogenetic switches are typically based on the Yeast Two-Hybrid system (Y2H) (Fields and Song, 1989), where light triggers photoreceptor self-dimerization, protein-protein interaction between different photoreceptors (i.e heterodimerization), or interaction between the photoreceptor and its interacting partner, which then result in activation of target gene expression (Salinas et al., 2017; Figueroa et al., 2021). For instance, the yLightOn optogenetic switch is based on self-dimerization, through the LOV (Light-Oxygen and Voltage) domain, of the blue-light photoreceptor Vivid (VVD) from *Neurospora crassa*, which has been used to enable light-mediated activation of the yeast cell cycle (Xu et al., 2018). Similarly, the OptoEXP, OptoINVT, and OptoAMP systems are based on self-dimerization of the EL222, a LOV-containing blue-light photoreceptor from *Erythrobacter litoralis*, which has been used to control the yeast metabolism and production of valuable metabolites upon light stimulation (Zhao et al., 2018; Zhao et al., 2020; Zhao et al., 2021).

Heterodimerization of blue-light photoreceptors has been also used in yeast optogenetics (Salinas et al., 2018). For instance, the FUN-LOV (FUNgal Light-Oxygen-Voltage) optogenetic switch is based on Y2H and the light-mediated interaction of LOV domains from the *N. crassa* blue-light photoreceptors WC-1 and VVD (Salinas et al., 2018). This switch has been used for light-dependent transcriptional activation of different genes, including the luciferase reporter gene (*Luc*), flocculation-associated genes (*FLO1* and *FLO11*), and the heterologous protein limonene synthase (Salinas et al., 2018). Recently, we have updated the FUN-LOV switch to place its components under the control of a strong promoter (*TDH3*) in low copy number plasmids (pRS313 and pRS315) (Romero et al., 2021). This optimization led to a FUN-LOV variant termed FUN-LOV^LS^ (Low copy number and Strong promoter), which resulted in a 10-fold increase in the levels of light-mediated target gene activation (Romero et al., 2021). One limitation of these systems, however, is that the components are encoded in two different plasmids, both of which require auxotrophic selection in the laboratory strain BY4741 (Salinas et al., 2018; Romero et al., 2021). Furthermore, the plasmids encoding the FUN-LOV system contain the same promoter and terminator sequences, restricting its genome integration by homologous recombination, and compromising the genetic stability of the system in the absence of auxotrophic selection (Salinas et al., 2018). Therefore, while the FUN-LOV system represents a powerful tool, various elements may limit its potential use for new applications in wild or industrial yeast strains.

In this work, we report a molecular redesign of FUN-LOV, encoding its components in a single plasmid (FUN-LOV^SP^), using different promoter and terminator sequences, and including the nourseothricin (NatMx) and hygromycin (HphMx) antibiotic resistances as selectable markers. These FUN-LOV variants (FUN-LOV^SP-Nat^ and FUN-LOV^SP-Hph^) showed similar levels of light-activated gene expression of the luciferase reporter compared to the FUN-LOV^LS^ and higher than the original FUN-LOV system. Furthermore, we integrate the FUN-LOV^SP-Nat^ and FUN-LOV^SP-Hph^ variants into the *HO* locus of the BY4741 strain, confirming the feasibility of stable and functional integration in the yeast genome. Finally, as proof of concept, we evaluate the functionality of the FUN-LOV^SP-Hph^ variant in the 59A-EC1118 wine yeast strain, showing similar levels of reporter gene induction upon blue-light stimulation compared to the laboratory strain, and with a lower background expression in darkness condition. Overall, we have reduced the molecular biology limitations of FUN-LOV, expanding its potential applications to wild and industrial yeast strains.

## 2 Materials and methods

### 2.1 Yeast strains and culture conditions

Two *S. cerevisiae* strains derived from the BY4741 genetic background (*MATa; his3∆1; leu2∆0; met15∆0; ura3∆0, gal3∆::KanMxRv-P*_*GAL1*_ *-Luc or P*_*5XGAL1*_ *-Luc*) were used in the experiments with FUN-LOV, FUN-LOV^LS^, FUN-LOV^SP^, FUN-LOV^SP-Nat^, and FUN-LOV^SP-Hph^. These strains carry the firefly luciferase reporter (*Luc*) gene controlled by either *GAL1* promoter or a *5XGAL1* synthetic promoter (five repetitions of the Gal4 Upstream Activating Sequence) integrated into the *GAL3* locus (Salinas et al., 2018). The 59A-EC1118 wine yeast strain was used for transformation and genome integration of the FUN-LOV^SP-Hph^ variant into the *HO* locus. Furthermore, the *Luc* reporter regulated by the *5XGAL1* promoter was integrated into the *GAL3* locus of the 59A-EC1118 strain. This strain is a haploid derivative from the EC1118 commercial wine strain previously described (Ambroset et al., 2011). The strains were maintained in YPDA medium (2% glucose, 2% peptone, 1% yeast extract, and 2% agar) at 30°C. For yeast genome integration of FUN-LOV^SP-Nat^ and FUN-LOV^SP-Hph^, the YPDA medium was supplemented with 300 μg/ml of nourseothricin and 100 μg/ml of hygromycin, respectively. The yeast strains used and developed in this work are listed in the Supplementary Table S1.

### 2.2 Design and generation of genetic constructs

The FUN-LOV^SP^ variant was designed *in silico* using the Geneious Prime software version 1.1 (Biomatters, New Zealand) and the online molecular biology platform Benchling (https://www.benchling.com/). The FUN-LOV^SP^ (3927 bp) variant was then synthetized using the Genewiz synthesis service (Genewiz, United States ). The synthetic FUN-LOV^SP^ was PCR amplified and cloned into the pRS316 and pRS426 plasmids using Yeast Recombinational Cloning (YRC) according to (Oldenburg et al., 1997). The FUN-LOV^SP-Nat^ and FUN-LOV^SP-Hph^ were constructed by PCR amplification of the FUN-LOV^SP^ and the nourseothricin (NatMx) or hygromycin (HphMx) antibiotic resistances, respectively, cloning both PCR fragments into the pRS316 plasmid through YRC. Briefly, the PCR products were amplified with Phusion Flash High-Fidelity PCR Master Mix (ThermoFisher Scientific, United States ), using primers with 50 bp of overhangs between adjacent PCR fragments (Oldenburg et al., 1997). These PCR products were co-transformed with the linearized target plasmid into the BY4741 strain using the standard Lithium acetate transformation protocol (Gietz and Schiestl, 2007). The assembled plasmids were then transferred to *E. coli* and confirmed by standard colony PCR. Finally, the genetic constructs were confirmed by Sanger sequencing (Macrogen Inc., Republic of Korea). Integration of the FUN-LOV^SP-Nat^ and FUN-LOV^SP-Hph^ variants into the genome of the BY4741 strain was carried out by direct PCR amplification, transformation, and recombination with the *HO* locus, using primers with 50 bp of overhang to the target locus. A similar procedure was carried out for genome integration of FUN-LOV^SP-Hph^ into the *HO* locus of the 59A-EC1118 wine yeast strain. Correct integration was confirmed by standard colony PCR using primers upstream and downstream the target locus. Plasmids and primers used and generated in this work are listed in Supplementary Tables S2, S3.

### 2.3 Luciferase expression and growth curves

Luciferase (*Luc*) was used as the reporter gene for light-activated gene expression (Salinas et al., 2018; Romero et al., 2021). Briefly, we used the destabilized version of the firefly luciferase, which has been optimized for real-time measurements of gene expression in yeast (Rienzo et al., 2012). The yeast strains carrying the *Luc* reporter and the different FUN-LOV variants were grown overnight in a 96-well plate format containing 200 µL of SC medium at 30°C. The next day, 20 µL of the overnight cultures were transferred to a 96-well plate with optical bottom (Nunc, ThermoFisher Scientific, United States ), containing 280 µL of SC media supplemented with 1 mM of luciferin. This plate was incubated at 25°C in a Synergy H1 microplate reader (Biotek, United States ) for simultaneous acquisition of Optical Density at 600 nm (OD_600_) and Luminescence (Lum) from each well every 10 min for 24 h (Romero et al., 2021). The *Luc* experiments were performed in three conditions: constant darkness (DD), constant blue-light illumination (BL), and a single blue-light pulse (BLP) of 2 h (h) duration (Romero et al., 2021; Rojas et al., 2022). In the DD assays, the 96-well plate was incubated inside the plate reader (dark) at 25°C with data acquisition of Lum and OD_600_ every 10 min. In the BL and BLP assays, the plate reader was programmed in a discontinuous kinetics protocol using the Gene5 software version 3.11 (Biotek, United States ). In the BL assays, the 96-well plate was incubated outside the plate reader (room temperature, 25°C) during 24-h for blue light illumination, inserting the 96-well plate automatically inside the equipment every 10 min for data acquisition of Lum and OD_600_. In the BLP assays, the 96-well plate was incubated inside the plate reader during 7-h (dark) at 25°C, then the 96-well plate was incubated outside of the equipment for blue light illumination during 2-h at room temperature (25°C), after the illumination, the 96-well plate was incubated again inside the equipment for 15-h. Data acquisition of Lum and OD_600_ in the BLP assays was performed every 10 min. The BL and BLP assays were performed using a LED blue-light illumination system developed by our research group (Supplementary Figure S1), which provide blue light at 466 nm with 24 μmol m^−2^ s^−1^ of light intensity, as was previously described (Romero et al., 2021). All the *Luc* expression assays were carried out in six biological replicates. The raw data of *Luc* expression measured as Lum in arbitrary units (a.u.) and the OD_600_ of the yeast cultures were graphed and used to generate the Supplementary Figures S2–S6. Based on these data sets, we normalized the luciferase expression dividing the Lum by the OD_600_ of the yeast cell cultures (Lum/OD_600 nm_), as described (Salinas et al., 2018).

### 2.4 Data analysis

The normalized *Luc* expression levels were used to compare the different FUN-LOV variants under a single BLP of 2-h duration (Romero et al., 2021). Furthermore, the average normalized *Luc* expression under DD condition and the fold-induction of *Luc* expression were also compared among FUN-LOV variants. The fold induction for each FUN-LOV variant was calculated according to the following equation:

$$Fold\;induction=\frac{Maximum\;normalized\;luminescence\;\left(\frac{Lum}{OD}\right)\;in\;the\;BLP\;condition}{average\;normalized\;luminescence\;\left(\frac{Lum}{OD}\right)\;in\;the\;DD\;condition}$$

Three parameters were statistically compared for each FUN-LOV variant respect to the original FUN-LOV system: 1) the maximal peak of *Luc* expression under BLP condition, 2) the average normalized *Luc* expression under DD condition, and 3) fold-induction of *Luc* expression for the *GAL1* and *5XGAL1* promoters. Statistical comparisons were carried out using GraphPad Prism version 9.4.0, performing one-way ANOVA with Kruskal-Wallis multiple comparisons test.

## 3 Results

### 3.1 Molecular redesign of the FUN-LOV system

**FIGURE 1:**
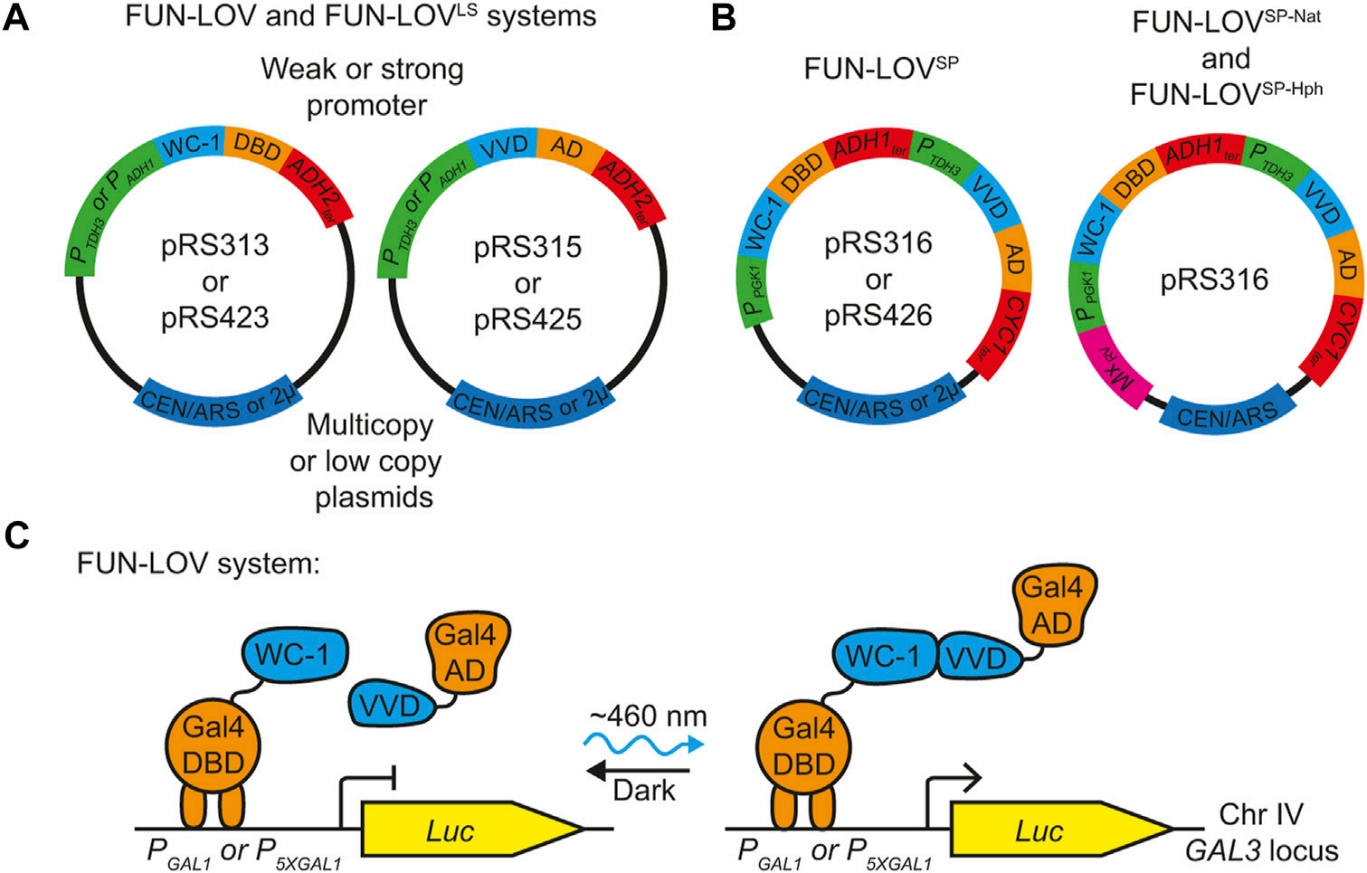
Molecular configuration of the FUN-LOV variants. **(A)** Plasmids encoding the original FUN-LOV switch. This system is encoded in two multicopy plasmids (pRS423 and pRS425), using the *ADH1* promoter (*P*_*ADH1*_) and *ADH2* terminator (*ADH2*_*ter*_) for all the components. The FUN-LOV^LS^ variant is encoded in two low-copy number plasmids (pRS313 and pRS315), and *P*_*ADH1*_ was replaced by the *TDH3* promoter (*P*_*TDH3*_). **(B)** The new FUN-LOV variants. The FUN-LOV^SP^ system is encoded in a single multicopy plasmid (pRS426) or a single low-copy plasmid (pRS316). In addition, the FUN-LOV^SP-Nat^ and FUN-LOV^SP-Hph^ variants are carrying the nourseothricin and hygromycin antibiotic resistances in the reverse orientation (MxRv), respectively. **(C)** The original FUN-LOV system architecture. The Gal4 DNA-Binding Domain (DBD) is linked to the LOV domain of WC-1, and the Gal4-activation domain (AD) is tethered to the LOV domain of VVD. Under blue light, the interaction between the LOV domains of both proteins reconstructs the Gal4 transcription factor, resulting in the expression of luciferase (*Luc*). The *Luc* gene is integrated into the yeast genome (Chr IV) and is controlled by *GAL1* (*P*_*GAL1*_) or 5XGAL1 (*P*_*5XGAL1*_) promoter.

The components of the original FUN-LOV system and its variant FUN-LOV^LS^ are encoded on two different plasmids, where both plasmids include the same promoter and terminator sequences (Figure 1A). This, makes genome integration of the systems difficult, limiting its application in wild and industrial yeast strains. Thus, we performed a rational molecular redesign and simplification of the original FUN-LOV system considering three elements: 1), the system must be encoded in a single plasmid to reduce plasmid burden (Karim et al., 2013); 2), the system should be devoid of repetitive elements such as the same promoter and terminator sequences for all components, facilitating its genome integration by homologous recombination; and 3), it must include an antibiotic resistance gene for selection after genome integration. The redesigned system, termed FUN-LOV^SP^ (Single Plasmid) and described here, was synthesized, and cloned into the pRS316 (low copy number) or into pRS426 plasmid (high copy number) (Figure 1B). The FUN-LOV^SP^ variant include two strong promoters, *PGK1* (*P*_*PGK1*_) and *P*_*TDH3*_, and two terminators, *ADH1* (*ADH1*_*ter*_) and *CYC1* (*CYC1*_*ter*_) for its different components (Figure 1B). We also developed two versions that includes the nourseothricin (NatMx) and hygromycin (HphMx) antibiotic resistance cassettes in reverse direction to avoid polymerase collisions with *P*_*PGK1*_ (Figure 1B). These variants were named FUN-LOV^SP-Nat^ and FUN-LOV^SP-Hph^, and considering that, we previously demonstrated that combining strong promoters with low copy number plasmids improves FUN-LOV performance (Romero et al., 2021), we cloned the FUN-LOV^SP-Nat^ and FUN-LOV^SP-Hph^ into the pRS316 plasmid (Figure 1B). Therefore, we generated three new FUN-LOV variants that reduce the molecular biology limitations of the original system.

**FIGURE 2:**
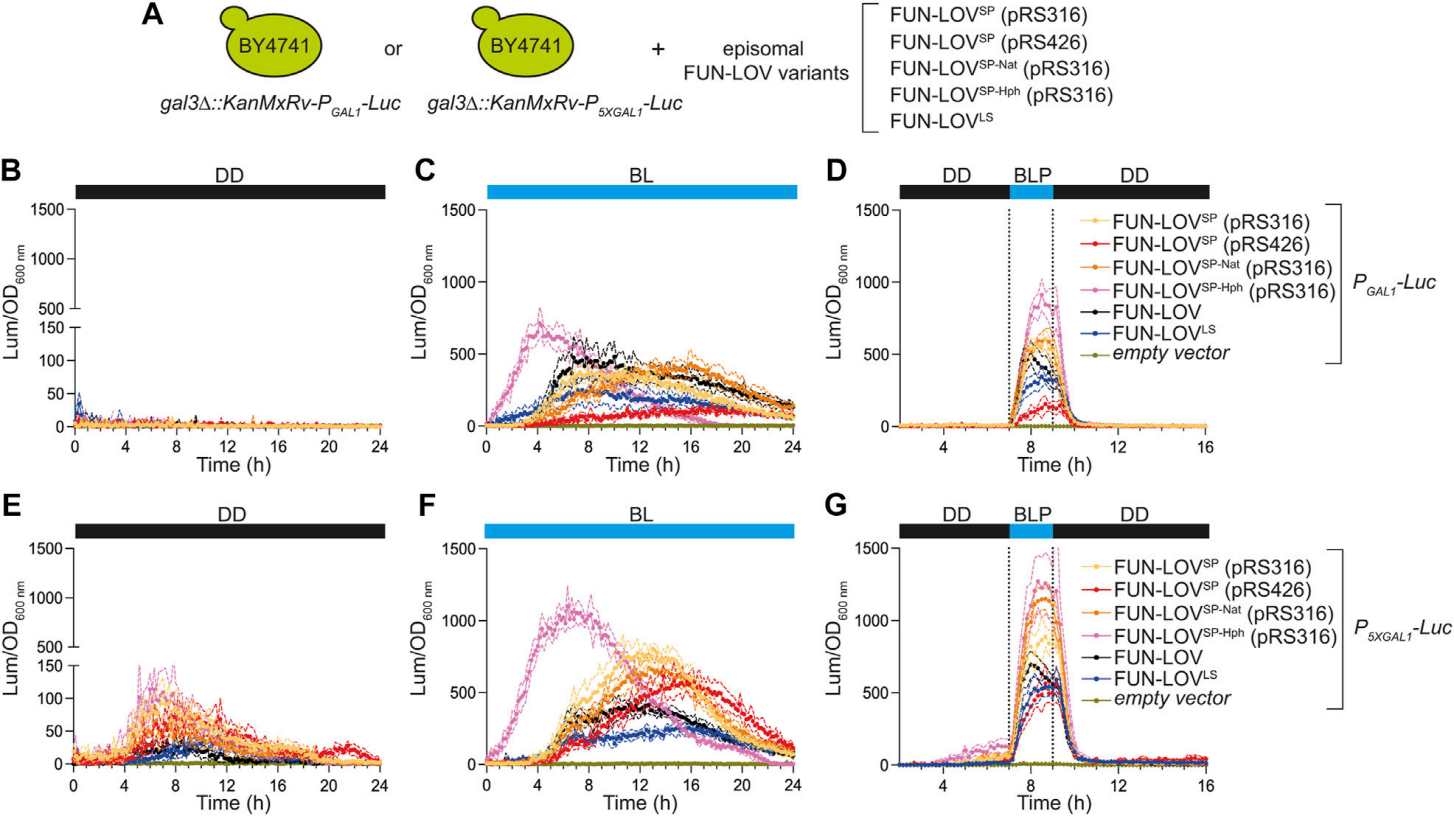
Luciferase expression in the BY4741 strains carrying different FUN-LOV variants episomally. **(A)** BY4741 yeast strains carrying the luciferase reporter gene (*Luc*) integrated into the genome and controlled by either the *GAL1* (*P*_*GAL1*_) or the 5XGAL1 (*P*_*5XGAL1*_) promoters. These strains were transformed with different FUN-LOV variants episomally to evaluate the impact of these systems in light-regulated reporter gene expression. **(B–G)** The luciferase expression was measured as luminescence (Lum) and normalized by the Optical Density at 600 nm (OD_600 nm_) of the corresponding yeast cell culture. Three experimental conditions were assayed: **(B,E)** constant darkness (DD), **(C,F)** constant blue-light (BL), and **(D, G)** a single blue-light pulse (BLP) of 2-h duration (dotted lines). In all panels, the average of six biological replicates is shown, with the standard deviation represented by dashed lines of the corresponding color. **(B–D)** assays with the strain expressing *Luc* under *P*_*GAL1*_. **(E–G)** assays with strains expressing *Luc* under *P*_*5XGAL1*_. The BY4741 yeast strain with the pRS316 plasmid not encoding the optogenetic system was used as basal luminescence level (*empty vector*).

The different FUN-LOV variants do not affect the Y2H-like architecture of the system, which is based on the light-mediated interaction of WC-1 and VVD (Figure 1C). Therefore, we expected the FUN-LOV^SP^, FUN-LOV^SP-Nat^, and FUN-LOV^SP-Hph^ to show similar levels of light-dependent reporter gene expression to the FUN-LOV and FUN-LOV^LS^ systems. To evaluate this, we compared the levels of light-controlled gene expression that can be achieved with each of the different FUN-LOV variants, using the luciferase (*Luc*) reporter regulated by the *GAL1* promoter (*P*_*GAL1*_) or the *5XGAL1* (*P*_*5XGAL1*_) synthetic promoter, and integrated into the *GAL3* locus (Salinas et al., 2018). Importantly, we selected a genome-integrated reporter gene to avoid a copy number variation effect on *Luc* expression. Each FUN-LOV variant was transformed episomally in the yeast strains carrying the *Luc* reporter (Figure 2A), and *Luc* expression was assayed under three experimental conditions: constant darkness (DD), constant blue-light (BL), and a single blue-light pulse (BLP) of 2-h duration. As expected, we observed that all the FUN-LOV variants resulted in low background levels of *Luc* expression in DD (Figures 2B,E). In contrast, under BL (Figures 2C,F) or BLP conditions (Figures 2D,G), high levels of *Luc* expression were observed. The background *Luc* expression in DD was higher for the *P*_*5XGAL1*_ promoter (compare Figures 2B,E), a phenomenon previously reported for this synthetic promoter (Salinas et al., 2018). Similarly, the *Luc* expression levels under BL and BLP were higher when the FUN-LOV variants targeted the *P*_*5XGAL1*_ promoter compared to the *P*_*GAL1*_ promoter (Figure 2). Remarkably, the FUN-LOV^SP^, FUN-LOV^SP-Nat^, FUN-LOV^SP-Hph^ variants, in the context of a low copy number plasmid (pRS316), showed equivalent or higher levels of *Luc* expression under BL and BLP conditions than FUN-LOV and FUN-LOV^LS^ systems (Figure 2). Interestingly, the FUN-LOV^SP^ in the context of a high copy number plasmid (pRS426) exhibited growth defects (Supplementary Figures S2, S3), confirming our previous observation that expression of FUN-LOV using strong promoters in a high copy number plasmid impairs yeast growth (Romero et al., 2021). In addition, we observed differences in the 24-h kinetics of normalized *Luc* expression among FUN-LOV variants under BL condition (Figures 2C,F), which are explained by differences in the yeast growth curves (Supplementary Figures S2, S3). In conclusion, the molecular redesign of FUN-LOV described here (FUN-LOV^SP^, FUN-LOV^SP-Nat^, and FUN-LOV^SP-Hph^ variants) increases *Luc* expression upon light stimulation.

### 3.2 FUN-LOV variants show expanded functionality

**FIGURE 3:**
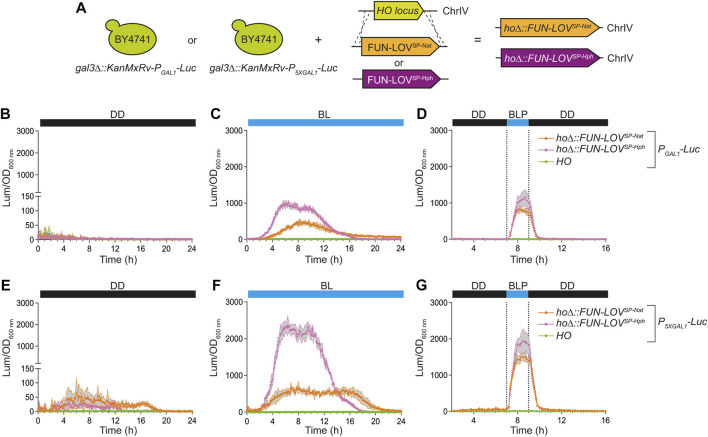
Luciferase expression in the BY4741 yeast strains carrying the FUN-LOV^SP-Nat^ and FUN-LOV^SP-Hph^ variants integrated into the genome. **(A)** BY4741 yeast strains carrying the luciferase reporter gene (*Luc*) integrated into the genome and controlled by *GAL1* (*P*_*GAL1*_) or 5XGAL1 (*P*_*5XGAL1*_) promoters. In these strains the FUN-LOV^SP-Nat^ and FUN-LOV^SP-Hph^ variants were integrated into the *HO* locus. **(B–G)** The luciferase expression was measured as luminescence (Lum) and normalized by the Optical Density at 600 nm (OD_600 nm_) of the corresponding yeast cell culture. Three illumination conditions were assayed: **(B,E)** constant darkness (DD), **(C,F)** constant blue-light (BL), and **(D,G)** a single 2-h blue-light pulse (BLP) (dotted lines). In all panels, the average of six biological replicates is shown, with the standard deviation represented by a shaded region. **(B–D)** assays with the strain expressing *Luc* under *P*_*GAL1*_. **(E–G)** assays with strains expressing *Luc* under *P*_*5XGAL1*_.

Potential applications of FUN-LOV^SP-Nat^ and FUN-LOV^SP-Hph^ in wild or industrial yeast isolates, where auxotrophic selection is not available, depend on the capacity to integrate these variants into the yeast genome. Initially, we used the yeast strains carrying the *Luc* reporter to integrate the FUN-LOV^SP-Nat^ and FUN-LOV^SP-Hph^ into the *HO* locus (Figure 3A). We selected the *HO* locus for genome integration since its deletion do not affect the yeast growth (Baganz et al., 1997). In the reporter strains, we measured *Luc* expression levels under DD, BL, and BLP conditions, confirming that genome integrated copy of the FUN-LOV^SP-Nat^ and FUN-LOV^SP-Hph^ variants are functional (Figure 3; and Supplementary Figures S4, S5). The genome-integrated copy of FUN-LOV^SP-Nat^ and FUN-LOV^SP-Hph^ resulted in comparable levels of *Luc* expression to those observed with FUN-LOV^SP-Nat^ and FUN-LOV^SP-Hph^ cloned into a low-copy plasmid (pRS316) under BL and BLP conditions (compare Figures 2, 3). Interestingly, the genome integrated FUN-LOV^SP-Hph^ variant resulted in higher *Luc* expression levels compared to the FUN-LOV^SP-Nat^ variant in the BL and BLP conditions (Figure 3). The HphMx and NatMx cassettes included in the FUN-LOV^SP-Hph^ and FUN-LOV^SP-Nat^ variants contain the same promoter and terminator sequences (*TEF*); where the open reading frames of *hph* and *nat1* have a high difference in the GC content, 58.7% and 70.7%, respectively (Goldstein and McCusker, 1999). This may explain the different levels of light-mediated gene expression of the *Luc* reporter obtained with the FUN-LOV^SP-Hph^ and FUN-LOV^SP-Nat^ variants (Figure 3). In addition, the FUN-LOV^SP-Nat^ and FUN-LOV^SP-Hph^ variants showed a different 24-h kinetics of *Luc* expression in BL condition (Figures 3C,F), which is due to differences in the growth curves between yeast strains (Supplementary Figures S4, S5). Therefore, the FUN-LOV^SP-Nat^ and FUN-LOV^SP-Hph^ variants can be integrated into the yeast genome and result in similar levels of target gene expression compared to episomally maintained copies, a finding that can simplify the implementation of this switch in multiple applications.

**FIGURE 4:**
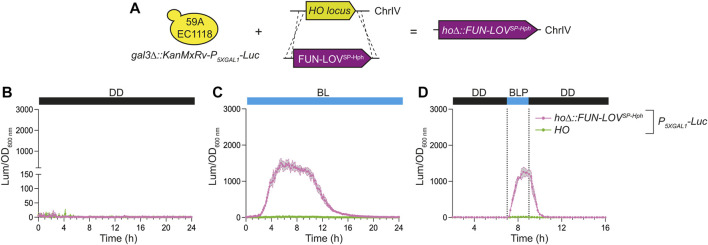
Luciferase expression in the 59A-EC1118 wine yeast strains carrying the FUN-LOV^SP-Hph^ variant integrated into the genome. **(A)** The 59A-EC1118 wine yeast strain is carrying the luciferase reporter gene (*Luc*) integrated into the genome and controlled by the *5XGAL1* (*P*_*5XGAL1*_) promoter. In this strain, the FUN-LOV^SP-Hph^ variant was integrated into the *HO* locus. **(B,C)** The luciferase expression was measured as luminescence (Lum) and normalized by the Optical Density at 600 nm (OD_600 nm_) of the corresponding yeast cell culture. Three illumination conditions were assayed: **(B)** constant darkness (DD), **(C)** constant blue-light (BL), and **(D)** a single 2-h blue-light pulse (BLP) (dotted lines). In all panels, the average of six biological replicates is shown, with the standard deviation represented by a shaded region.

As a proof of concept to demonstrate the applicability of the new FUN-LOV variants in a different genetic background, we demonstrated the FUN-LOV^SP-Hph^ functionality in the 59A-EC1118 wine yeast strain. Initially, we inserted the *Luc* reporter controlled by the *P*_*5XGAL1*_ into the *GAL3* locus as target gene to assess the FUN-LOV^SP-Hph^ functionality. Then, we integrated the FUN-LOV^SP-Hph^ into the *HO* locus of the 59A-EC1118 strain (Figure 4A). The 59A-EC1118 strain carrying the integrated copy of FUN-LOV^SP-Hph^ was assayed under DD, BL, and BLP conditions (Figure 4 and Supplementary Figures S6). The results demonstrated that FUN-LOV^SP-Hph^ is functional in the 59A-EC1118 wine yeast strain, showing high levels of *Luc* expression in BL and BLP conditions, with lower *Luc* expression in DD (Figure 4). Under BL and BLP conditions the *Luc* expression levels in the 59A-EC1118 strain resulted in comparable levels to those observed in the BY4741 strain (compare Figures 3, 4). Interestingly, under DD condition, 59A-EC1118 strain showed a lower *Luc* expression background respect to the BY4741 strain with the *P*_*5XGAL1*_ (Figures 3E, 4B), suggesting differences in the galactose genes regulation between these yeast strains. In conclusion, the FUN-LOV^SP-Hph^ variant is functional in a wine yeast strain, confirming that we reduced the molecular biology limitations of the original system, and expanding the potential application of this optogenetic tool beyond the laboratory strain.

### 3.3 Comparing transcriptional activation capacity among FUN-LOV variants

**FIGURE 5:**
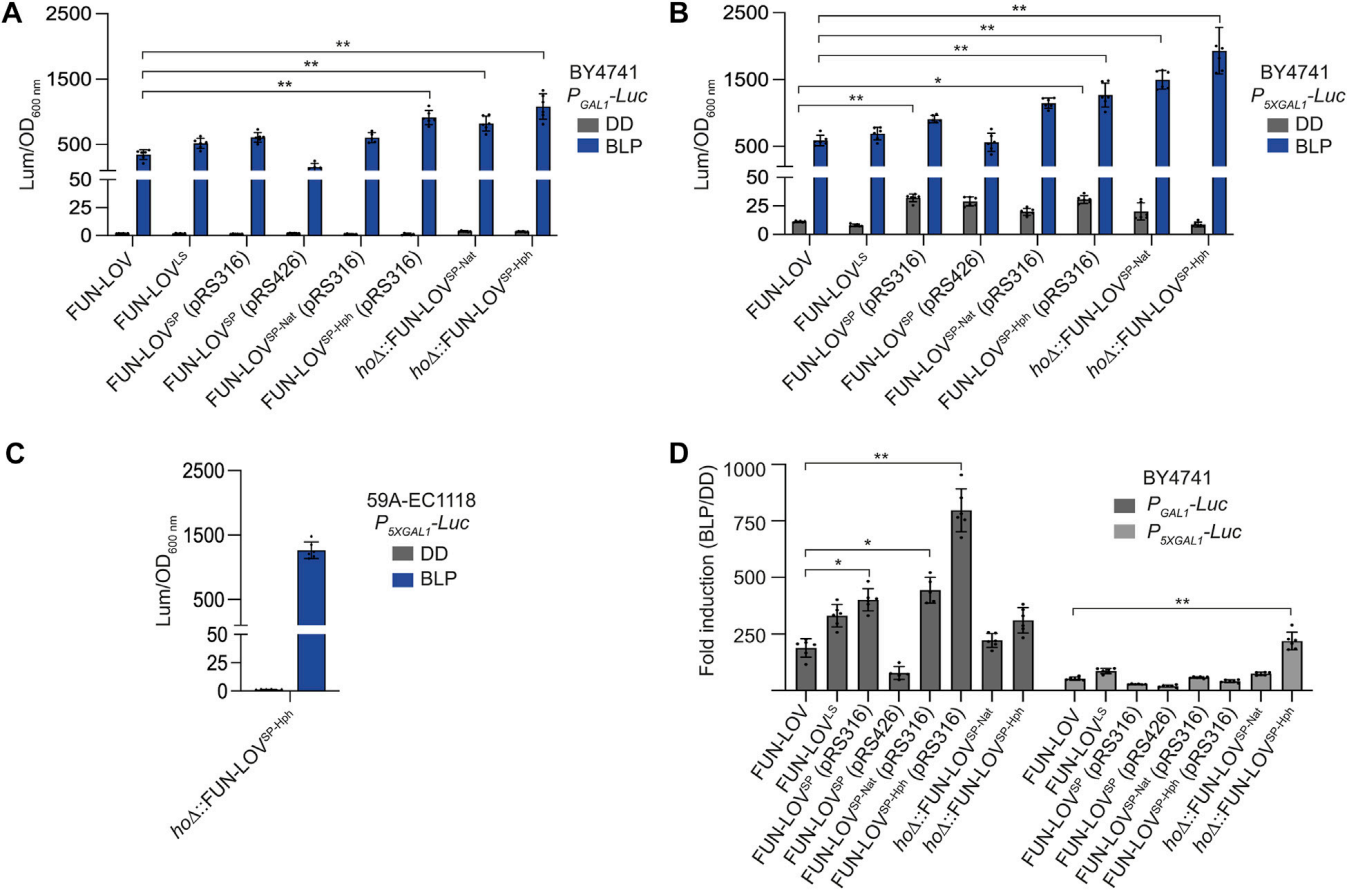
Dynamic range for different FUN-LOV variants. **(A,B)** Maximal normalized luciferase expression for each FUN-LOV variant upon a single 2-h blue-light pulse (BLP) and its average background expression in constant darkness condition (DD). Results for the BY4741 yeast strains carrying the luciferase reporter (*Luc*) gene controlled by *P*_*GAL1*_ **(A)** or *P*_*5XGAL1*_ **(B)** promoters are shown. **(C)** Maximal normalized luciferase expression for the FUN-LOV^SP-Hph^ variant upon a single 2-h blue-light pulse (BLP) and its average background expression in constant darkness condition (DD). Results for the 59A-EC1118 wine yeast strains carrying the *Luc* reporter gene controlled by *5XGAL1* promoter (*P*_*5XGAL1*_) promoter is shown. **(D)** Fold induction of luciferase expression (BLP/DD) controlled by *P*_*GAL1*_ and *P*_*5XGAL1*_ promoters in the BY4741 strains carrying different FUN-LOV variants. The asterisk (*p < 0.05) and double asterisk (** *p* < 0.01) represents statistically significant differences using One-way ANOVA. In all panels, the average of six biological replicates with the standard deviation is shown.

We compared the maximal level of *Luc* expression for each FUN-LOV variant respect to the original FUN-LOV system using the same illumination conditions: a single BLP of 2-h duration (see methods for details). Interestingly, the low-copy versions of FUN-LOV^SP^, FUN-LOV^SP-Nat^, and FUN-LOV^SP-Hph^ variants showed higher levels of maximal *Luc* expression upon a single BLP compared to FUN-LOV and FUN-LOV^SP^ (Figure 5A,B). Importantly, the copy of FUN-LOV^SP-Nat^ and FUN-LOV^SP-Hph^ integrated into the *HO* locus did not decrease the maximal luciferase expression, showing a similar behavior compared to the FUN-LOV^SP-Nat^ and FUN-LOV^SP-Hph^ encoded in the pRS316 plasmid (Figures 5A,B). In the BY4741 strain with the *Luc* reporter controlled by the *P*_*5XGAL1*_, the FUN-LOV^SP-Nat^ and FUN-LOV^SP-Hph^ variants resulted in higher *Luc* expression levels under the BLP condition compared to the BY4741 strain with *Luc* reporter regulated by *P*_*GAL1*_ (Figures 5A,B), but also showed a higher background of *Luc* expression in DD (Figure 5B). Interestingly, in the 59A-EC1118 strain with the *Luc* reporter controlled by the *P*_*5XGAL1*_, the *Luc* expression levels in DD and BLP conditions for FUN-LOV^SP-Hph^ are comparable to those observed in the BY4741 strain with the *Luc* reporter regulated by the *P*_*GAL1*_ (Figures 5A,C). Thus, the results indicate that in the 59A-EC1118 genetic background the *P*_*5XGAL1*_ behaves as the canonical *P*_*GAL1*_.

We then calculated the fold induction of each system, dividing the maximal *Luc* expression in BLP condition by the average background of *Luc* expression in DD (see methods). As a result, we observed that FUN-LOV^SP-Hph^ encoded in pRS316 plasmid is the system with higher fold-induction of *Luc* expression when *P*_*GAL1*_ is used (Figure 5D). Importantly, in all the FUN-LOV variants, the *Luc* fold-induction strongly decreased in the BY4741 yeast strain carrying the luciferase reporter controlled by the *P*_*5XGAL1*_ (Figure 5D). This is due to the higher *Luc* expression background observed in DD for the *P*_*5XGAL1*_ compared to *P*_*GAL1*_ (Figures 5A,B), a phenomenon previously reported for *P*_*5XGAL1*_ (Salinas et al., 2018). Interestingly, the *Luc* fold-induction calculated in the 59A-EC1118 wine yeast strain (FUN-LOV^SP-Hph^ + *P*_*5XGAL1*_ *-Luc*) is 982-fold, which is comparable to the fold-induction observed in the BY4741 strain with the FUN-LOV^SP-Hph^ variant and *Luc* reporter regulated by the *P*_*GAL1*_ (Figure 5D). Altogether, the FUN-LOV variants developed in this work reached higher levels of *Luc* expression upon a single BLP and higher fold-induction of *Luc* expression compared to the previously described versions, increasing the dynamic range of this optogenetic system.

## 4 Discussion

The original FUN-LOV optogenetic switch is encoded in two different multicopy plasmids (Salinas et al., 2018). Recently, the FUN-LOV switch was subjected to a molecular optimization, whereby the system is encoded in two low copy number plasmids and its expression is controlled by the *TDH3* promoter, in a variant known as FUN-LOV^LS^ (Romero et al., 2021). This variant results in a 10-fold increase in the levels of luciferase expression upon blue-light stimulation. These experiments, however, were carried out using the FUN-LOV^LS^ and the luciferase reporter encoded in multicopy plasmids (Romero et al., 2021), which can result in copy number variation and genetic instability, and thus, in high variability. In this work, we aimed to avoid copy number variation by using two strains where the luciferase reporter has been integrated into the yeast genome (Salinas et al., 2018). Importantly, in the FUN-LOV variants developed here (FUN-LOV^SP^, FUN-LOV^SP-Nat^, and FUN-LOV^SP-Hph^), the complete optogenetic system is encoded in a single plasmid (SP) of low copy number (pRS316). In addition, we integrated the FUN-LOV^SP-Nat^ and FUN-LOV^SP-Hph^ variants into the yeast genome and demonstrated its functionality and showing that can result in higher levels of luciferase expression than to the original system (Figure 5). Interestingly, the FUN-LOV switch and its variants showed a response to blue-light during the exponential growth phase, which begins at 4 h of yeast growth approximatively (Supplementary Figures S2–S6). This observation is related to the transcriptional activity of the promoters (*ADH1*, *TDH3*, and *PGK1*) used to control the FUN-LOV components expression. These promoters are active during the glucose consuming phase, reducing its activity in stationary phase or when glucose has been depleted (Partow et al., 2010; Salinas et al., 2018). This suggest that the chimeric proteins of the FUN-LOV system are available for light response only during the exponential growth phase. In conclusion, our results of light-dependent *Luc* expression are consistent with the growth kinetics of each yeast strain.

Other optogenetic systems based on blue-light photoreceptors have been integrated into the yeast genome, reporting similar levels of a reporter gene expression compared to the genome integrated FUN-LOV^SP-Nat^ and FUN-LOV^SP-Hph^ variants (Zhao et al., 2018; Zhao et al., 2020). For instance, the OptoEXP and OptoINVRT optogenetic systems have been integrated into the yeast genome, using the *HIS3* locus or ∂-sites (Zhao et al., 2018; Zhao et al., 2020). Similarly, the optogenetic system based on CRY2-CIB1 interaction has been integrated into the *URA3* locus (An-Adirekkun et al., 2020). Importantly, the fold-induction of luciferase expression obtained by FUN-LOV^SP-Nat^ and FUN-LOV^SP-Hph^ variants are comparable to the yLightOn system (573-fold), an optogenetic switch described in yeast and based on VVD self-dimerization (Xu et al., 2018). Beyond blue-light optogenetic systems, the fold-induction for luciferase expression achieved by the FUN-LOV variants is comparable to those previously reported for optogenetic systems based on red-light photoreceptors in yeast. For instance, the optogenetic system based on the red-light photoreceptor Phytochrome B (PhyB) and its interacting protein PIF3, both from *Arabidopsis thaliana*, achieved a 1000-fold of induction for the *lacZ* reporter (Shimizu-Sato et al., 2002). Similarly, the optogenetic system based on the red light-dependent interaction of the Phytochrome A (PhyA) photoreceptor and FHY1 protein, also both from *A. thaliana*, permitted a 300-fold of induction for a luciferase reporter (Sorokina et al., 2009). Therefore, the levels of gene expression achieved by the FUN-LOV variants developed in this work are higher than the original FUN-LOV system, and similar to other optogenetic systems described in yeast.

As proof of concept to demonstrate the applicability of our FUN-LOV variants, we implemented the FUN-LOV^SP-Hph^ variant in the 59A-EC1118 wine yeast strain, showing to be fully functional in this genetic background. This is the first demonstration of a functional optogenetic switch in a wine yeast strain, opening the possibility for optogenetic control of oenological phenotypes such as glycerol, acetate, and ethanol production. Furthermore, the FUN-LOV variants described here could be used to control the microbial interactions of the yeast community present at the beginning of the fermentation process (Walker and Pretorius, 2022). In addition, the FUN-LOV variants developed in this work could be also used in wine strains for functional characterization of horizontally acquired genes, which have been described as key players in yeast adaptation to different environmental conditions (Devia et al., 2020). Therefore, the FUN-LOV variants reported in this work have multiple applications in wine yeast research.

In conclusion, we have expanded the FUN-LOV variants, encoding the system in a single plasmid (FUN-LOV^SP^), and including antibiotic resistances (FUN-LOV^SP-Nat^ and FUN-LOV^SP-Hph^) that enables its genome integration in two different genetic backgrounds (BY4741 and 59A-EC1118). These variants reduced the molecular biology limitations of FUN-LOV, promoting its future applications in wild and industrial strains to control biotechnologically relevant phenotypes by light.

## Data Availability

The original contributions presented in the study are included in the article/Supplementary Material, further inquiries can be directed to the corresponding author.
