# Supplementary material for: Expanding the molecular versatility of an optogenetic switch in yeast
Source: Front Bioeng Biotechnol. 2022 Nov 15;10:1029217. doi: 10.3389/fbioe.2022.1029217 (PMC9705753; doi:10.3389/fbioe.2022.1029217)
Supplement: Supplementary file 1 [file DataSheet1.pdf]

Supplementary Material

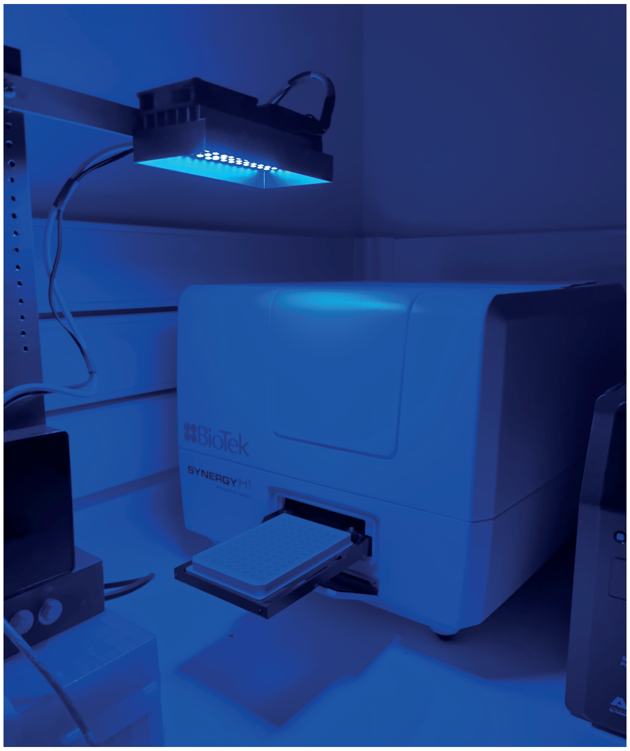

Supplementary Figure S1.Illumination system used in our experiments. The illumination system includes 128 RGB LED lights, which provide blue light at 466 nm as described (Romero et al., 2021). In the discontinuous kinetics for the BL (blue light) and BLP (blue light pulse) experiments, the 96-well plate is illuminated outside of the equipment at room temperature (25 ºC) and inserted automatically into the equipmet every 10 min for data acquisition of luminescence and OD at 600 nm (see methods).

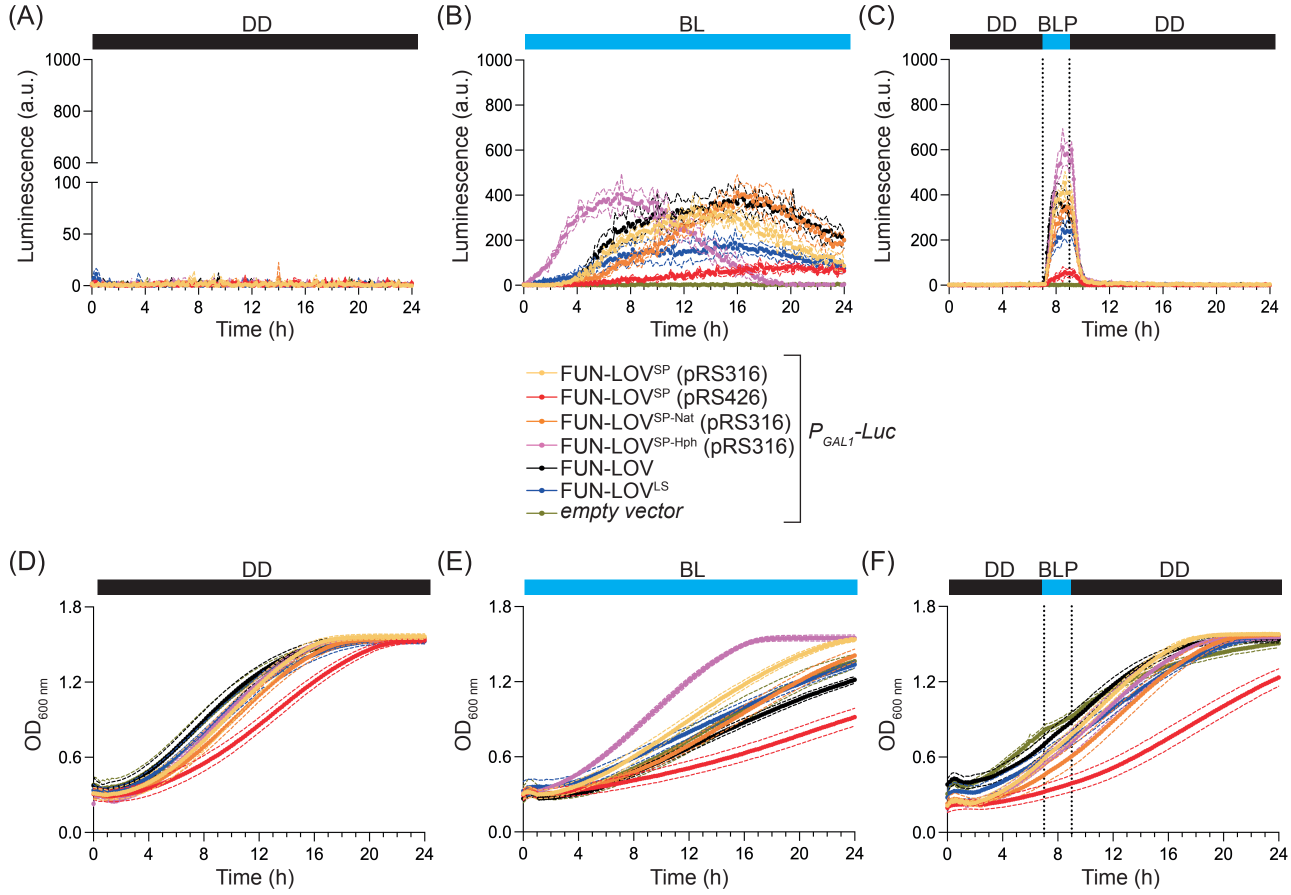

Supplementary Figure S2. Raw data for luciferase expression and growth curves in BY4741 yeast strains carrying different FUN-LOV variants apisomally. The luciferase expression (panels A, B and C) measured as luminescence in arbitrary units (a.u.) and the Optical Density (OD) at 600 nm (panels D, E, and F) of the yeast cultures are shown. The luciferase expression is controlled by the *GAL1* promoter (P_{GAL1}), integrated in the *GAL3* locus, and measured under three different experimental conditions: (A and D) constant darkness (DD), (B and E) constant blue-light (BL), and (C and F) a single blue-light pulse (BLP) of 2h (dotted lines). In all panels, the average of six biological replicates is shown, with the standard deviation represented by color dashed lines. The BY4741 yeast strain with the pRS316 plasmid not encoding the optogenetic system was used as basal luminescence level (*empty vector*). In the main text, Figure 2B, C, and D were generated using this data set.

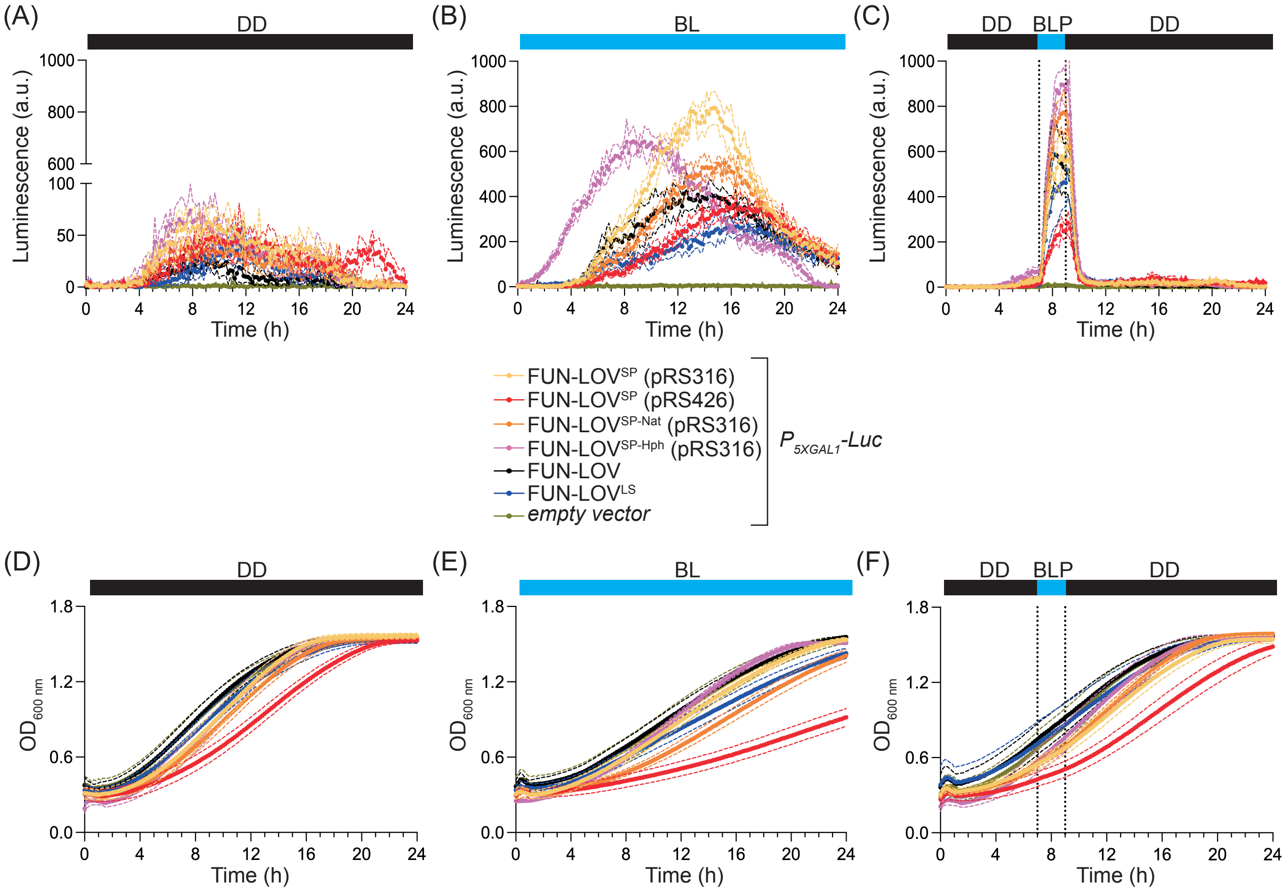

Supplementary Figure S3. Raw data for luciferase expression and growth curves in the BY4741 yeast strain carrying different FUN-LOV variants episomally. The luciferase expression (panels A, B and C) measured as luminescence in arbitrary units (a.u.) and the Optical Density (OD) at 600 nm (panels D, E, and F) of the yeast cultures are shown. The luciferase expression is controlled by the synthetic *5XGAL1* promoter (P_{5XGAL1}), integrated in the *GAL3* locus, and measured under three different experimental conditions: (A and D) constant darkness (DD), (B and E) constant blue-light (BL), and (C and F) a single blue-light pulse (BLP) of 2h (dotted lines). In all panels, the average of six biological replicates is shown, with the standard deviation represented by colour dashed lines. The BY4741 yeast strain with the pRS316 plasmid not encoding the optogenetic system was used as basal luminescence level (*empty vector*). In the main text, Figure 2E, F, and G were generated using this data set.

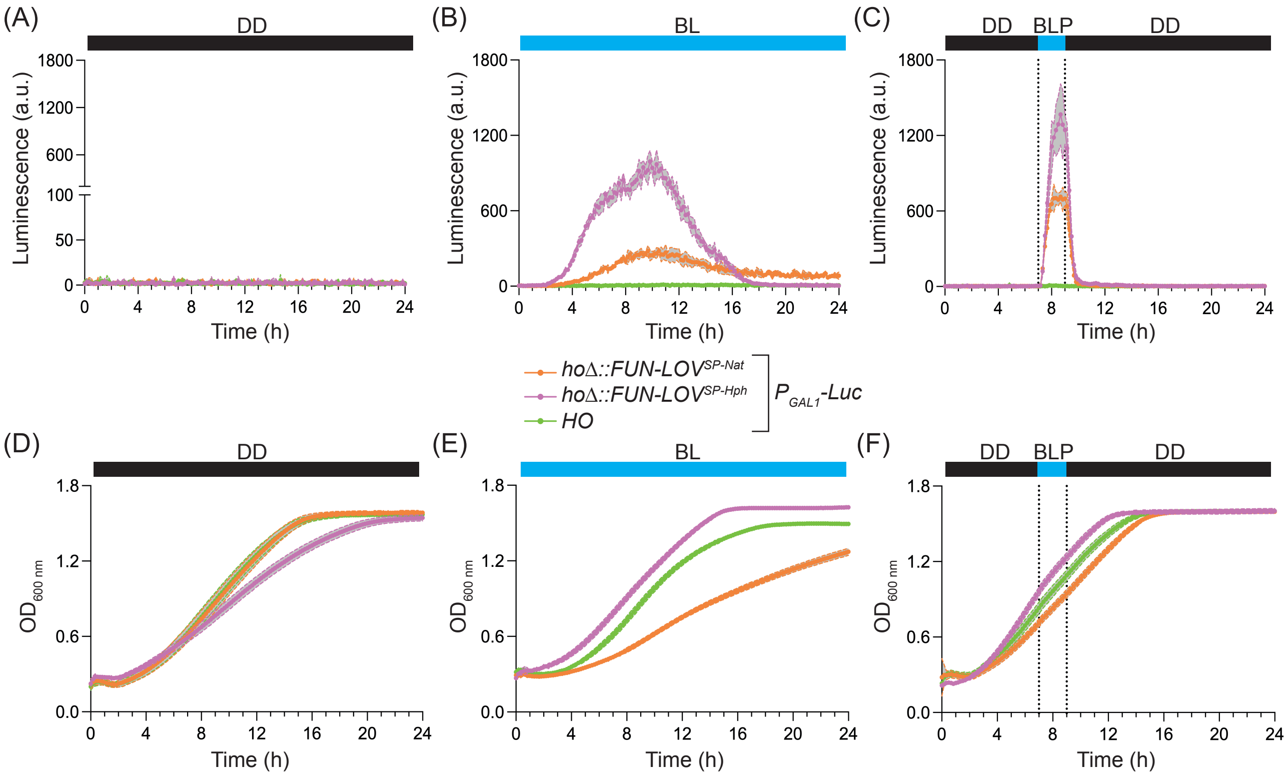

Supplementary Figure S4. Raw data for luciferase expression and growth curves in the BY4741 yeast strain carrying the genome integrated FUN-LOV$^{SP\text{-}Nat}$ and FUN-LOV$^{SP\text{-}Hph}$ variants. The luciferase expression (panels A, B and C) measured as luminescence in arbitrary units (a.u.) and the Optical Density (OD) at 600 nm (panels D, E, and F) of the yeast cultures are shown. The luciferase expression is controlled by the *GAL1* promoter (P_{GAL1}), integrated in the *GAL3* locus, and measured under three different experimental conditions: (A and D) constant darkness (DD), (B and E) constant blue-light (BL), and (C and F) a single blue-light pulse (BLP) of 2h (dotted lines). In all panels, the average of six biological replicates is shown, with the standard deviation represented as shaded region. In the main text, Figure 3B, C, and D were generated using this data set.

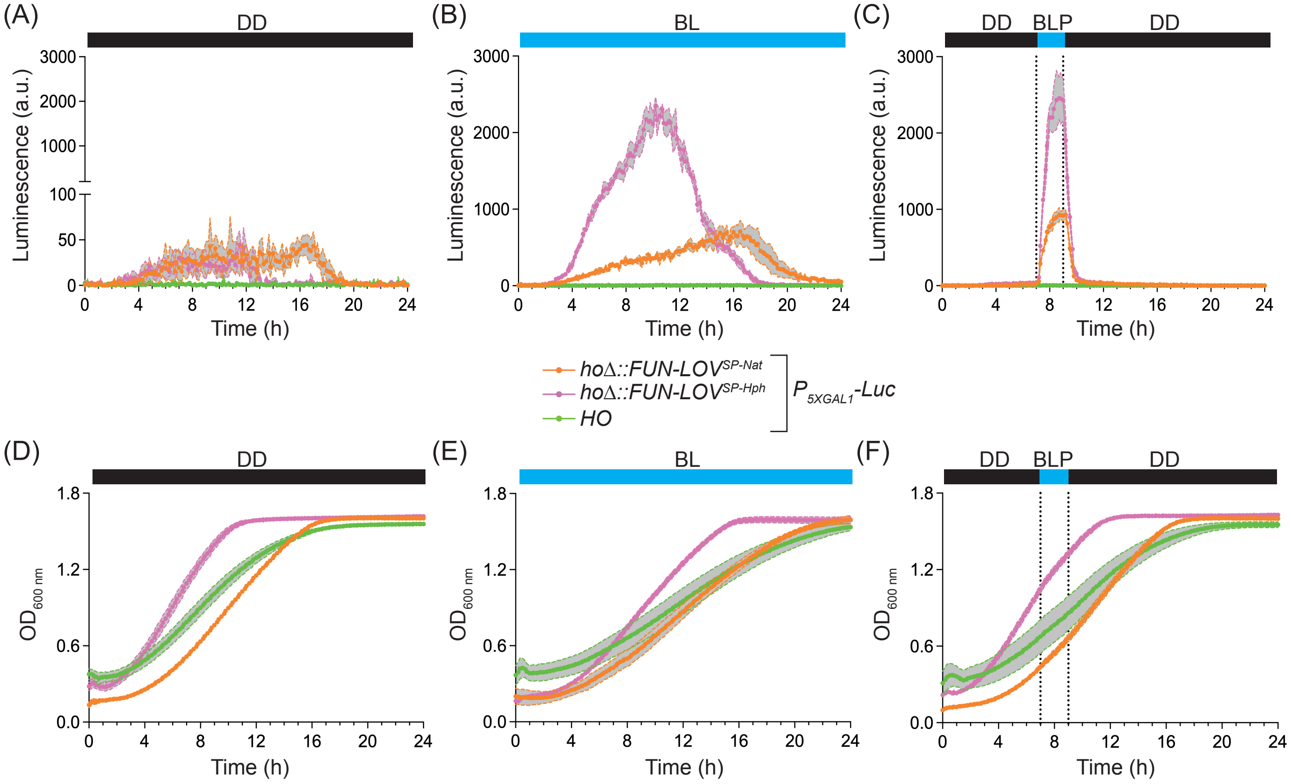

Supplementary Figure S5. Raw data for luciferase expression and growth curves in the BY4741 yeast strain carrying the genome integrated FUN-LOV$^{SP\text{-}Nat}$ and FUN-LOV$^{SP\text{-}Hph}$ variants. The luciferase expression (panels A, B and C) measured as luminescence in arbitrary units (a.u.) and the Optical Density (OD) at 600 nm (panels D, E, and F) of the yeast cultures are shown. The luciferase expression is controlled by the synthetic *5XGAL1* promoter (P_{5XAL1}), integrated in the *GAL3* locus, and measured under three different experimental conditions: (A and D) constant darkness (DD), (B and E) constant blue-light (BL), and (C and F) a single blue-light pulse (BLP) of 2h (dotted lines). In all panels, the average of six biological replicates is shown, with the standard deviation represented as shaded region. In the main text, Figure 3E, F, and G were generated using this data set.

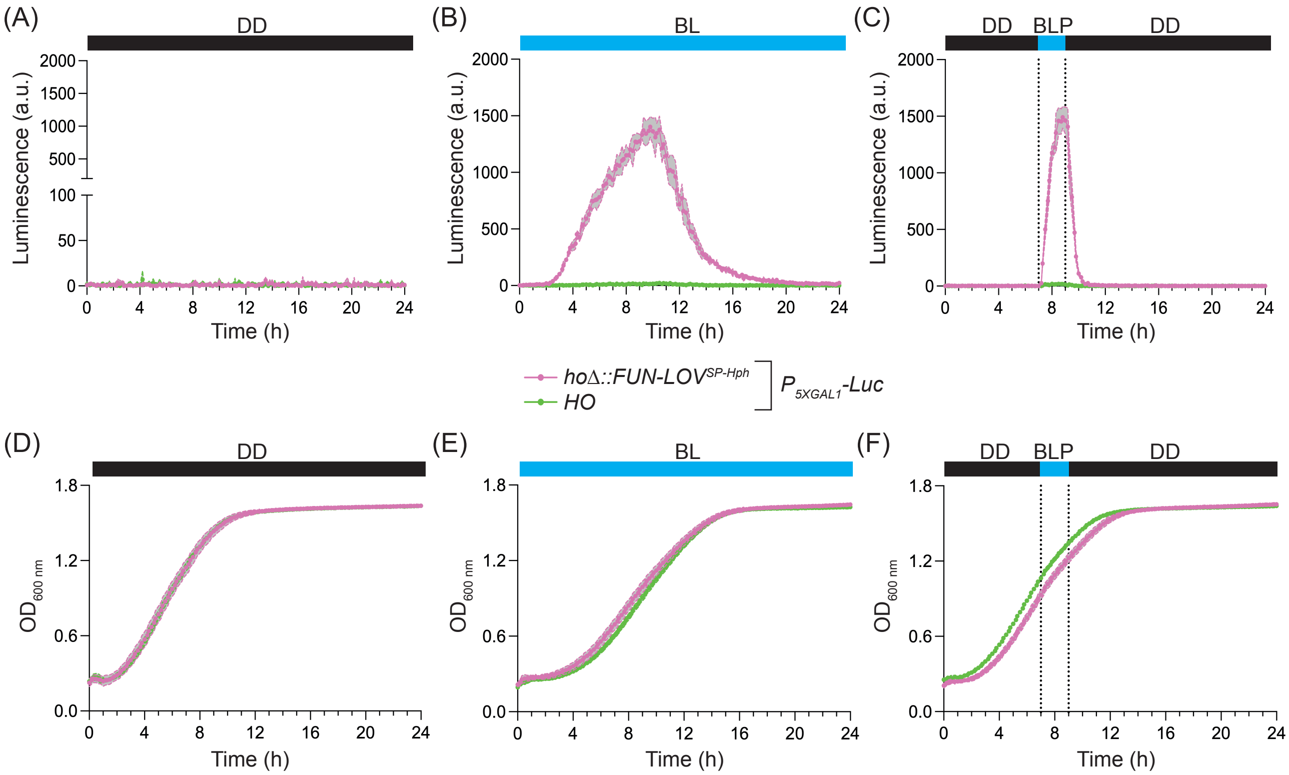

Supplementary Figure S6. Raw data for luciferase expression and growth curves in the 59A-EC1118 wine yeast strain carrying the genome integrated FUN-LOV^{SP-Hph} variant. The luciferase expression (panels A, B and C) measured as luminescence in arbitrary units (a.u.) and the Optical Density (OD) at 600 nm (panels D, E, and F) of the yeast cultures are shown. The luciferase expression is controlled by the synthetic *5XGAL1* promoter (P_{5XAL1}), integrated in the *GAL3* locus, and measured under three different experimental conditions: (A and D) constant darkness (DD), (B and E) constant blue-light (BL), and (C and F) a single blue-light pulse (BLP) of 2h (dotted lines). In all panels, the average of six biological replicates is shown, with the standard deviation represented as shaded region. In the main text, Figure 4B, C, and D were generated using this data set.

Supplementary Table S1. *S. cerevisiae* strains used and developed in this work.

Strain Name	Genotype*	Source
BY4741	*MATa his3Δ1 leu2Δ0 met15Δ0 ura3Δ0*	Euroscarf
59A-EC1118	*MATa, ho*	Ambroset et al., 2011
Y32	BY4741; *gal3Δ::KanMxRv-*P_{GAL1}*-Luc*	Salinas et al., 2018
Y33	BY4741; *gal3Δ::KanMxRv-*P_{5XGAL1}*-Luc*	Salinas et al., 2018
Y32 FUN-LOV	Y32; plasmids 1 and 2	Salinas et al., 2018
Y32 FUN-LOV	Y33; plasmids 1 and 2	Salinas et al., 2018
Y196	Y32; plasmid pRS316	This work
Y197	Y32; plasmid 6	This work
Y197	Y33; plasmid pRS316	This work
Y198	Y33; plasmid 6	This work
Y235	Y32; plasmid 7	This work
Y236	Y33; plasmid 7	This work
Y268	Y32; plasmid pRS426	This work
Y270	Y33; plasmids 3 and 4	This work
Y271	Y32; plasmids 3 and 4	This work
Y301	Y32; plasmids pRS313 and pRS315	This work
Y302	Y33; plasmids pRS423 and pRS425	This work
Y306	Y33; *hoΔ::FUN-*$LOV^{SP\text{-}Nat}$	This work
Y307	Y32; *hoΔ::FUN-*$LOV^{SP\text{-}Nat}$	This work
Y333	Y32; plasmid 8	This work
Y334	Y33; plasmid 8	This work
Y335	59A-EC1118, *gal3Δ::KanMxRv-*P_{5XGAL1}*-Luc*	This work
Y337	Y335, *hoΔ::FUN-*$LOV^{SP\text{-}Hph}$	This work
Y343	Y32, *hoΔ::FUN-*$LOV^{SP\text{-}Hph}$	This work
Y344	Y33, *hoΔ::FUN-*$LOV^{SP\text{-}Hph}$	This work

*Plasmids information in Supplementary Table S2

Supplementary Table S2. Plasmids used and generated in this work.

Plasmid number	Name	Genetic construct	Vector backbone	Reference
1	WC1_423 (FUN-LOV)	*pADH1-WC1-GAL4 DBD-ADH2*$_{ter}$	pRS423	Salinas et al., 2018
2	VVD_425 (FUN-LOV)	*pADH1-VVD-GAL4 AD-ADH2*$_{ter}$	pRS425	Salinas et al., 2018
3	WC1_313 (FUN-LOV^{LS})	*pTDH3-WC1-GAL4 DBD-ADH2*$_{ter}$	pRS313	Romero et al., 2021
4	VVD_315 (FUN-LOV^{LS})	*pTDH3-VVD-GAL4 AD-ADH2*$_{ter}$	pRS315	Romero et al., 2021
5	FUN-LOV^{SP}	*pPGK1-WC1-GAL4 DBD-ADH1*$_{ter}$ *pTDH3-VVD-GAL4 AD-CYC1*$_{ter}$	pRS316	This work
6	FUN-LOV^{SP}	*pPGK1-WC1-GAL4 DBD-ADH1*$_{ter}$ *pTDH3-GAL4 AD-VVD-GAL4 AD-CYC1*$_{ter}$	pRS426	This work
7	FUN-LOV^{SP-Nat}	*NatMxRv-pPGK1-WC1-GAL4 DBD-ADH1*$_{ter}$ *pTDH3-VVD-GAL4 AD-CYC1*$_{ter}$	pRS316	This work
8	FUN-LOV^{SP-Hph}	*HphMxRv-pPGK1-WC1-GAL4 DBD-ADH1*$_{ter}$ *pTDH3-VVD-GAL4 AD-CYC1*$_{ter}$	pRS316	This work

Supplementary Table S3. Primers used in this work.

Name	Sequence (5'-3')	Length (nt)	Orientation	Description
oL172	GGTAACGCCAGGGTTTTCCCAGTCACGACGTGGATCCTTGCAAATTAAAG	50	Rv	Cloning FUN-LOV^{SP}, FUN-LOV$^{SP\text{-}Nat}$, and FUN-LOV$^{SP\text{-}Hph}$ in pRS426 or pRS316.
oL256	ATGAAATCGCCATGCCAAGC	20	Fw	Amplification of *KanMxRv-*P_{GAL1}*-Luc* and *KanMxRv-*P_{5XGAL1}*-Luc* from Y32 and Y33 strains, respectively; and recombination into the *GAL3* locus.
oL257	GTGCGGAGCCACTCTGACTC	20	Rv	Amplification of *KanMxRv-*P_{GAL1}*-Luc* and *KanMxRv-*P_{5XGAL1}*-Luc* from Y32 and Y33 strains, respectively; and recombination into the *GAL3* locus.
oL291	AGCGGATAACAATTTCACACAGGAAACAGCTGGATCCTTGCAAATTAAAG	50	Rv	Cloning FUN-LOV^{SP} in pRS426.
oL307	AAACAGATCTGGCGCGCCTTAATTAACCCGGTGAGTAAGGAAAGAGTGAG	50	Fw	Assembly of FUN-LOV^{SP} with NatMx or HphMx.
oL308	GCGATAGTTCCTCACTCTTTCCTTACTCACCGGGTTAATTAAGGCGCGCC	50	Rv	Assembly of FUN-LOV^{SP} with NatMx or HphMx.
oL307	TTGTAATACGACTCACTATAGGGCGAATTGATCGATGAATTCGAGCTCGT	50	Fw	Cloning FUN-LOV$^{SP\text{-}Nat}$ and FUN-LOV$^{SP\text{-}Hph}$ in pRS316.
oL336	TCTAAATCCATATCCTCATAAGCAGCAATCAATTCTATCTATACTTTAAAatcgatgaattcgagctcgt	70	Fw	FUN-LOV$^{SP\text{-}Nat}$ and FUN-LOV$^{SP\text{-}Hph}$ amplification and recombination into the *HO* locus.
oL337	ATTAAATTTTACTTTTATTACATACAACTTTTTAAACTAATATACACATTtggatccttgcaaattaaag	70	Rv	FUN-LOV$^{SP\text{-}Nat}$ and FUN-LOV$^{SP\text{-}Hph}$ amplification and recombination into the *HO* locus.
oL340	GAATTGTACTACCGCTGGGC	20	Fw	Confirmation of FUN-LOV$^{SP\text{-}Nat}$ and FUN-LOV$^{SP\text{-}Hph}$ *HO* genome integration.
oL341	TGGTTGAAACAAATCAGTGCCG	22	Rv	Confirmation of FUN-LOV$^{SP\text{-}Nat}$ and FUN-LOV$^{SP\text{-}Hph}$ *HO* genome integration.
